# Determination of the electronic portal imaging device pixel‐sensitivity‐map for quality assurance applications. Part 1: Comparison of methods

**DOI:** 10.1002/acm2.13603

**Published:** 2022-04-15

**Authors:** Michael Paul Barnes, Baozhou Sun, Brad Michael Oborn, Bishnu Lamichhane, Stuart Szwec, Matthew Schmidt, Bin Cai, Frederick Menk, Peter Greer

**Affiliations:** ^1^ Department of Radiation Oncology Calvary Mater Hospital Newcastle Newcastle New South Wales Australia; ^2^ School of Mathematical and Physical Sciences University of Newcastle Newcastle New South Wales Australia; ^3^ Department of Radiation Oncology Washington University in St. Louis St. Louis Missouri USA; ^4^ Centre for Medical Radiation Physics University of Wollongong Wollongong New South Wales Australia; ^5^ Illawarra Cancer Care Centre Wollongong Hospital Wollongong New South Wales Australia; ^6^ School of Medicine and Public Health University of Newcastle Newcastle New South Wales Australia

**Keywords:** EPID dosimetry, pixel‐sensitivity‐map (PSM), quality assurance (QA)

## Abstract

**Purpose:**

Calibration of a radiotherapy electronic portal imaging device (EPID) using the pixel‐sensitivity‐map (PSM) in place of the flood field correction improves the utility of the EPID for quality assurance applications. Multiple methods are available for determining the PSM and this study provides an evaluation to inform on which is superior.

**Methods:**

Three different empirical methods (“Calvary Mater Newcastle” [CMN], “Varian,” and “WashU”) and a Monte Carlo‐based method of PSM determination were investigated on a single Varian TrueBeam STx linear accelerator (linac) with an aS1200 EPID panel. PSM measurements were performed for each empirical method three successive times using the 6 MV beam. The resulting PSM from each method was compared to the Monte Carlo method as a reference using 2D percentage deviation maps and histograms plus crossplane profiles. The repeatability of generated PSMs was also assessed via 2D standard deviation (SD) maps and histograms. Additionally, the Beam‐Response generated by removal of the PSM from a raw EPID image for each method was visually contrasted. Finally, the practicality of each method was assessed qualitatively and via the measured time required to acquire and export the required images.

**Results:**

The median pixel‐by‐pixel percentage deviation between each of the empirical PSM methods and the Monte Carlo PSM was ‐0.36%, 0.24%, and 0.74% for the CMN, Varian, and WashU methods, respectively. Ninety‐five percent of pixels were found to be repeatable to within ‐0.21%, 0.08%, 0.19%, and 0.35% (1 SD) for the CMN, Monte Carlo, Varian, and WashU methods, respectively. The WashU method was found to be quickest for data acquisition and export and the CMN the slowest.

**Conclusion:**

For the first time four methods of generating the EPID PSM have been compared in detail and strengths and weaknesses of each method have been identified. All methods are considered likely to be clinically acceptable and with similar practical requirements.

## 1 INTRODUCTION

The required routine linear accelerator (linac) quality assurance (QA) tests and their tolerances and frequencies are stipulated in best practice documents such as the American Association of Physicists in Medicine Task Group 142 report^1^ and Medical Physics Practice Guideline 8.a.^2^ Both documents require routine QA testing of the beam dose profile shape and the photon beam quality for which a number of publications have suggested that metrics such as flatness and off‐axis‐ratio can be used as an effective measure.^3^, ^4^, ^5^

The amorphous silicon electronic portal imaging device (EPID) was designed for patient positioning applications. For such applications, the image non‐uniformities caused by the patient anatomy in the beam are of interest. As such, correcting out the non‐uniformities introduced by the beam and those introduced by the EPID panel itself are required. This is standardly achieved via the flood field calibration procedure. However, correcting out the non‐uniformities introduced by the beam is problematic for many linac and patient QA applications because the beam non‐uniformities (i.e., profile shape) is the information required for investigation. As such, the flood field correction represents a major current limiting factor to the use of EPID for dosimetry and QA applications.

Because of the flood field correction, the EPID‐based profile QA present in the literature^6^, ^7^, ^8^, ^9^ has generally been a constancy check relative to a baseline that was set once the system had been calibrated using an alternate device. The EPID cannot currently be used for evaluation of the absolute beam profile as this is removed by flood field calibration, and thus the user must apply different and less‐efficient QA methods. For patient QA the flood field correction is also problematic in that the EPID panel must not be translated and hence the extremities of asymmetric or off‐axis fields can miss the panel and not be recorded. This is particularly relevant for measurements of breast treatment fields. While several EPID dosimetry systems exist, the majority use non‐water equivalent dosimetry (i.e., EPID signal predictions) or if they estimate dose‐to‐water planes, the true beam profile is not reproduced, which means the EPID methods are not comparable to other detector systems with regards to their utility. Conversion of EPID to dose‐to‐water including beam profile variations would provide the convenience and high spatial resolution of EPID imaging while generating data in the general dose‐to‐water format similarly to the commercial array type devices. This would potentially be a game‐changer for the use of EPID for quality assurance applications including linac testing and commissioning, ongoing quality assurance as well as pretreatment and in vivo dosimetry.

Because of the problems associated with the EPID flood field calibration a number of authors have attempted to develop alternate EPID calibration procedures where the non‐uniformity of the imager response is corrected without disturbing the non‐uniformity of the incident beam.^10^, ^11^, ^12^, ^13^ Such a calibration procedure was first attempted by Greer^10^ who named this new calibration procedure the pixel‐sensitivity‐map (PSM). The PSM is analogous to the array type calibrations used in commercial 2D‐array detectors to correct for response differences between individual detectors.

Three studies have been published which examined the utilization of PSM‐corrected EPID images for linac QA purposes.^14^, ^15^, ^16^ The study of Yaddanapudi et al.^14^ utilized PSM‐corrected EPID images for linac acceptance testing purposes. The study used changes in the flatness of the beam profile as measured using PSM‐corrected EPID imaging as a measure of beam energy. This study also suggested that the method could be used for beam symmetry evaluation, although no assessment of symmetry was presented. The study of Cai et al.^15^ also looked at QA applications of PSM‐corrected EPID images. The focus of that study was to demonstrate that PSM‐corrected EPID imaging could provide consistent profiles for matched linacs. Both the Yaddanapudi and Cai studies were based upon an adaptation of the Boriano et al.^11^ method of determining the PSM. The study of Barnes et al.^16^ used PSM‐corrected EPID imaging as an absolute measure of wide‐field beam symmetry as a means of photon beam angle steering. The PSM used in the Barnes publication is based upon a simplified version of the Greer^10^ method of PSM correction.

Similarly to the linac QA applications, PSM calibration has demonstrated utility in EPID‐based patient‐specific QA applications.^17^ Unlike flood‐field‐corrected EPID images, correcting for the PSM would allow for first principles type dose‐to‐water conversion of EPID images that would allow for more direct comparison between patient QA EPID images and the treatment plans. The image would first be corrected by the PSM to remove the EPID introduced image non‐uniformities while preserving the profile shape, which can subsequently be converted to dose‐to‐water.

A PSM is currently only being used commercially in the Standard Imaging (Standard Imaging, Middleton, WI, USA) Adaptivo product.^18^ However, it is currently not clear whether the PSM method used in Adaptivo is optimal or whether there is a better method available.

It is the purpose of this study to evaluate several existing methods of determining the PSM; including an improved Greer method henceforth known as the Calvary Mater Newcastle (CMN) method, a modified Boriano method henceforth known as the WashU method, and a method developed by Varian (Varian Medical System, Palo Alto, CA, USA) henceforth known as the Varian method. A fourth method is also included based on data obtained from Monte Carlo simulations (the Monte Carlo method). Other methods of determining the PSM have been published in the literature,^12^, ^13^ but only the locally available methods are included in this study. The aim of the study is to evaluate each method and help identify which method of PSM determination is superior in terms of accuracy, efficiency, and usability so that the method of PSM determination can be standardized and used universally for QA applications using EPID. To the authors best knowledge, direct comparison between available PSM methods has not been published previously. Such a study is important to inform on the relative benefits and weaknesses of each method and hence determine which method is superior for individual applications so that PSM‐corrected imaging can be widely utilized.

Due to the substantial number of results presented, the study is separated into two parts, presented in separate publications. This publication constitutes part 1 and concentrates on introducing the PSM and methods of determining it followed by analysis from one linac and with one beam (6 MV). The second part, published separately, extends the analysis to other photon beams (i.e., 6 MV FFF, 10 MV, and 10 MV FFF) to inform on beam dependence of both the PSM itself and of the methods of determining it.

## 2 METHODS

In non‐flood‐field‐corrected (raw) EPID images there are non‐uniformities introduced from multiple sources. These non‐uniformities can generally be categorized as either introduced by the EPID imager or via the incident beam. When multiplied together, the former is referred to as the PSM and the latter, in this study, as the Beam‐Response. With this definition the PSM includes any source of non‐uniformity present when the EPID is irradiated with a hypothetical completely uniform beam. As such, the effect of EPID arm backscatter would be included in the PSM. However, it should be noted that the EPID arm backscatter is field size dependent^19^ meaning that a backscatter independent PSM would need to be derived, combined with backscatter correction of raw images. It should also be noted that the Beam‐Response, while analogous to a dose‐to‐water profile will not have the same shape. This is because the EPID panel is not water equivalent and there is an over‐response to the low energy spectrum in the silicon layer.^20^ This has the effect of increasing the magnitude of the beam horns compared to the dose‐to‐water beam profiles.

### 2.1 Materials

The measurements in this study were performed on a single Varian TrueBeam STx linac with aS1200 EPID. The aS1200 panel utilizes a 43 × 43 cm^2^ panel with 40 × 40 cm^2^ active area in an 1190 × 1190 pixel array used for dosimetry mode, resulting in resolution of 0.34 mm at isocenter. The aS1200 EPID also has a backscatter shielding plate to remove backscatter from the EPID support arm such that this source of non‐uniformity encapsulated in the PSM is minimized in this study. Comparison of results was performed using a custom Matlab script (Mathworks Inc., Natick, MA, USA).

### 2.2 Measurement methods

#### 2.2.1 Methods of PSM determination

In this study, four methods of determining the PSM were investigated.

##### Modified Greer method (CMN)

The methodology for measuring Beam‐Response at discrete points on the EPID panel is presented in Barnes et al.^16^ and the reader is referred to this publication and that of Greer^10^ for details of the basic method. The underlying concept is that the PSM can be isolated from the Beam‐Response by taking a series of images where the beam is kept constant (e.g., 5 × 5 cm^2^ field at central axis), but imaged with different parts of the EPID panel by offsetting the panel and hence with different pixel sensitivity. If an image acquired with offset EPID is divided by an image where the EPID is centered, then the Beam‐Responses will cancel and the variation from unity in the ratio image will be due solely to differences in the PSM. This assumes that the beam is consistent between irradiations. This assumption is minimized by using a relatively large MU (100 MU) per irradiation to minimize the effect of beam instability in the first few MUs. For this study, measurement points were acquired at 5 cm intervals in both lateral and longitudinal directions on the EPID panel to create a 6 × 7 array of data points ranging from ‐15 to +15 cm off‐axis in the lateral direction and from ‐15 to +10 cm off‐axis in the longitudinal direction. The dataset is limited in the longitudinal direction due to the inability to translate the EPID panel any further as discussed in Barnes et al.^16^ The 6 × 7 array of PSM data points is then removed from a raw (non‐flood field corrected) wide‐field EPID image to provide the Beam‐Response at the corresponding spatial points. Because the Beam‐Response is expected to be smooth like a dose profile then the 6 × 7 Beam‐Response array can be fitted with a 2D mathematical function for the full 40 × 40 cm^2^ image. The resulting 2D Beam‐Response fit can then be removed from the raw EPID image to provide the PSM for all pixels in the 1190 × 1190 EPID image.

The 2D Beam‐Response fitting is based upon cubic spline interpolation^21^, ^22^ to the given data set in the range of measurements. The cubic spline interpolation is applied using mathematical “not‐a‐knot” and symmetric boundary conditions. The cubic spline approach provides a smooth fit of the given function inside the interpolated region, and not‐a‐knot boundary conditions ensure that the third derivative of the interpolant matches at the boundary, resulting in a smooth fit overall. In the second step, a linear extrapolation approach is applied to extrapolate the function from the data points out to 20 cm. The linear extrapolation method was assessed as being the most appropriate form of extrapolation, but this is a potential weakness of the method, which is discussed in the results and discussion sections.

##### Modified Boriano method (WashU)

The WashU method is based upon the method of Boriano et al.,^11^ summarized by Cai et al.^15^ The reader is referred to these publications for details of the basic method. The concept was originally described by Simon et al.,^23^ who used the technique for ion chamber array calibrations. Boriano then applied the method to Elekta EPID calibrations with minor modifications and the medical physics team at Washington University subsequently applied it to Varian EPIDs. The principal concept of this method is to deliver several sets of large overlapping field irradiations to the EPID with small EPID shifts between each irradiation.^15^ These shifts result in the pixels in each image being subjected to slightly different parts of the beam. If the signals at the different pixels irradiated with the same part of the beam and hence the same Beam‐Response from the different images are compared, then the difference must be due solely to the different pixel sensitivities and different PSM. By applying a recursive algorithm^11^, ^24^ the method can be propagated and the pixel sensitivities across the whole panel determined.

To achieve best repeatability and avoid errors due to image lag and variation of fluence the overlapping field images were acquired with Alternating Beam and Dark Fields henceforth known as the ABDF technique.^15^ XML‐scripts (Varian TrueBeam Developer Mode 2.7) were developed to automate the entire acquisition process. During the beam‐on time, 2 MUs were delivered for each beam‐on image with modulated dose rate and synchronized acquisition to ensure that the maximum signal was derived without saturating the imager. The dark fields taken during the beam‐hold period were later subtracted from the raw images to eliminate the background noise and residual signal when radiation had ceased. This process was repeated 100 times. The first 40 pairs of images were discarded to avoid fluence from a previous irradiation field. From the remaining 60 pairs of images, the average image with beam and average image without beam was calculated. The advantage of this ABDF technique is in eliminating ghosting effects for each frame and therefore reproducing the true pixel signal per frame. Finally, a recursive algorithm was used to derive the full PSM.^15^

##### Varian method (Varian)

The Varian PSM procedure was developed by Varian Medical Systems, but is not currently commercially available. The method assumes no change in beam fluence for subsequent deliveries and any variation of beam fluence can lead to systematic error propagation. Similar to WashU method, the ABDF imaging acquisition technique was used. Three open fields with the largest field size were first delivered with EPID shifts along the lateral and longitudinal directions by 50 pixels to calculate an initial estimate of the sensitivity map using the previously mentioned PSM recursive algorithm. Two EPID images were then acquired at different vertical shifts (‐15 and ‐75 cm). The initial PSM was then applied to the two images by considering the inverse square correction. The initial PSM was corrected to minimize the difference of the two PSM‐corrected images and to generate the intermediate PSM. When finished, the two PSM‐corrected scaled images should be equal (except for the scaling). During Step 3, four small images were acquired with the same open field at four different quadrants of EPID. The four images were corrected using the intermediate PSM. The difference between any “small” images was fitted using a polynomial function for final tuning of the intermediate PSM. When done, the four small images should be equal after PSM correction.

##### Monte Carlo‐derived method (Monte Carlo)

A Monte Carlo approach can also be used to determine the PSM.^12^ In the current study a Monte Carlo model was created for the TrueBeam linac and aS1200 EPID to examine the Beam‐Response from a first‐principles approach, that is, examining the pure energy deposition within the gadolinium (Gd) layer of the EPID. The dosimetric assumption made is that the amount of light produced by the Gd layer, hence the EPID signal or reading, is directly proportional to the energy deposition in the Gd layer. This assumption is considered valid based upon the work of Blake et al.,^25^ who found that ignoring the optical blurring was found to be sufficient to predict EPID dose–response in most scenarios.^26^ For the scenario of relevance to this current study, that of profile shape, Blake et al.^25^ found no statistically different shape between optical and energy deposition profiles.

The Monte Carlo model was developed in two stages. The first stage was the generation of a flood field phase space file for the beam prior to the EPID. This was generated using BEAMnrc 2019.^27^ The parent X‐ray beam was modeled directly from the Varian‐provided phase space files of the TrueBeam linacs. The BEAMnrc simulation contained secondary collimator jaws, mirror, and 120 leaf Millenium Mulit‐Leaf‐Colimator (MLC). In order to produce the flood field phase space file, the MLCs were simulated in the retracted position and the jaws at a 40 × 40 cm^2^ field size. All particles were simulated from the TrueBeam phase space files (starting above the jaws) and transported to below the MLCs at the level of 442 mm above the isocenter level. These particles were stored in a secondary phase space file. Next, Geant4 version 10.6.p02^28^ was used to simulate the radiation transport through the EPID. All layers were implemented as per the vendor notes, and the dose was scored in the Gd layer at a resolution that matches the native EPID pixel resolution of 1190 × 1190 pixels over a 40 cm field of view, giving a 0.34 mm pixel size. The resulting dose represents the idealised EPID Beam‐Response and this was simulated for the 6 MV beam in this study.

At the native pixel resolution, the raw Monte Carlo energy deposition contains a high level of statistical noise (it was deemed unfeasible to run enough histories to obtain an error in the native pixels of even just ±2%). The simulation results also showed no significant radial asymmetry of the image response. This reflects several confirmed features of the radiation beams:

1. The vast majority of EPID dose comes from primary X‐rays generated within an almost circular spot size within the target.
2. The jaw scatter component (for the 40 × 40 cm flood image) is not significant.

Hence, it becomes possible to generate a smooth 1D off‐axis dose response function derived from the Monte Carlo data. In essence, pixels in concentric rings around the central EPID pixel can be averaged to determine the raw off‐axis Beam‐Response function. A simple averaging of the pixels in concentric rings spaced 5 mm apart was therefore used to generate a raw Beam‐Response profile, in dose per primary history (electrons hitting the X‐ray target). This approach provides an increasingly smoother response as the radius increases due to more pixels being used to record the average. However, toward the centre of the EPID array (smaller radius rings), there is much less signal to average and so the response is still noisy (also higher than 200 mm radius becomes the corners of the EPID panel so less signal in total). The final stage in the creation of the 1D radial fit was then to apply a smooth fit to the Monte Carlo‐derived data.

Once the Monte Carlo‐derived ideal Beam‐Response was calculated then this Beam‐Response was removed from a measured raw EPID image to produce the PSM for that particular EPID panel.

#### 2.2.2 Normalization

The PSM, by definition, is internally normalized. However, the empirical methods are not automatically normalized similarly. The inherent noise of the PSM means that consistent normalization between methods is required to avoid systematic variation that is not real as per the PSM definition of this study. To avoid this each determined PSM was renormalized in each instance to the mean of the central 10 × 10 pixels. The Beam‐Response is analogous to a dose profile for which the convention is to normalize to central axis. As such, renormalization to central axis was performed for each Beam‐Response measurement.

#### 2.2.3 Data collection

Data were collected across a single measurement session so that the PSM could be calculated for each of the four methods with the same beam and EPID characteristics. Data were collected three successive times using the 6 MV beam for each method to allow comparison and assessment of repeatability. The CMN and Monte Carlo methods directly result in the determination of the Beam‐Response while the Varian and WashU methods result in the PSM being directly determined. In the measurement session wide‐field EPID images were also taken for the 6 MV beam energy. The applied flood field for each image was removed to leave the raw EPID image. For the Monte Carlo and CMN methods, the measured Beam‐Response was removed from the raw image to provide the PSM, while for the WashU and Varian methods the determined PSM was removed from the raw images to provide the measured Beam‐Response. In this way, Beam‐Responses and PSM were calculated for each method for comparison.

In addition to the EPID images required for each PSM method, in each measurement session the time required to acquire all of the images and to export them was recorded.

#### 2.2.4 Comparison of methods

##### Comparison of PSM

For each empirical method the PSM was compared against the corresponding Monte Carlo‐derived PSM. There was no ground truth PSM available for reference so the Monte Carlo method was chosen to compare each of the empirical methods against. It is acknowledged that the Monte Carlo method has its own limitations and these are described in Sections 3 and 4. Results were presented as a combination of 2D percentage deviation maps (pixel‐by‐pixel) with percentage deviation histograms plus crossplane 1D profiles and percentage deviation profiles. The mean, median, and standard deviation (SD) were calculated for each percentage deviation histogram.

##### Comparison of Beam‐Response

For the Monte Carlo and CMN methods, the Beam‐Response is generated directly from the method. In the case of the WashU and Varian methods, the PSM is directly generated and the Beam‐Response is subsequently determined indirectly via removal of the generated PSM from the original raw image. From the generated Beam‐Responses from each of the four methods, central axis crossplane profiles were extracted, overlaid, and visually contrasted.

##### PSM short‐term repeatability

The repeatability of the PSM derived from each of the four methods was assessed by calculating an SD map and histogram for each of the methods from the three successive measurements. Repeatability is presented as 1 SD divided by the mean (%) for each pixel. In this analysis, the Monte Carlo method is a special case in that rather than three distinct Beam‐Responses being generated for repeatability comparison only a single Beam‐Response is generated. The generated Beam‐Response is subsequently removed from three successive raw EPID images to provide three successive PSMs for repeatability analysis. Hence, this analysis describes repeatability of the EPID image itself and provides a reference for comparing the repeatability of the empirical methods. The raw EPID image repeatability is theoretically influenced by changes in the PSM and changes in the incident beam and this analysis could be performed without removal of the Monte Carlo Beam‐Response. However, the analysis has been performed with the Monte Carlo Beam‐Response removed to provide visually comparable results for comparison with the empirical methods.

##### Practicality of methods

The practicality of each method was assessed qualitatively by considering the simplicity of the method and analysis including whether advanced skills or extra equipment or software is required. The time required to acquire and export the image data required for each method was recorded and compared.

## 3 RESULTS

**FIGURE 1:**
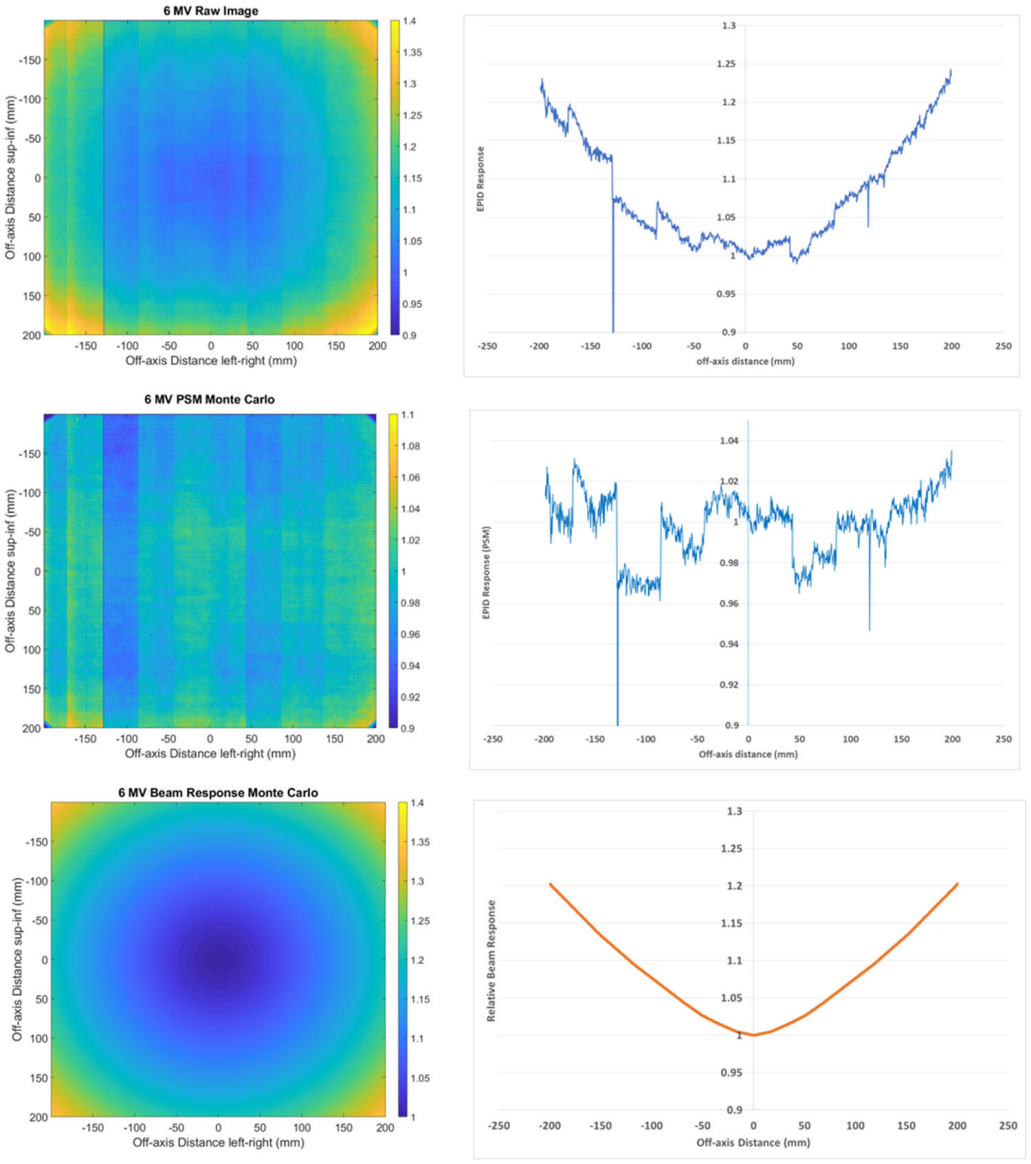
6 MV raw image, that is non‐flood field corrected (top), 6 MV Monte Carlo‐derived pixel‐sensitivity‐map (PSM) (middle), and 6 MV Monte Carlo‐derived Beam‐Response (bottom). Two‐dimensional (left) and 1D crossplane profile (right)

An example 6 MV raw EPID image (i.e., non‐flood field corrected) is presented in Figure 1a. As discussed this image can be separated into the EPID (PSM) (Figure 1b) and beam (Beam‐Response) (Figure 1c) introduced image non‐uniformities. In Figure 1, the 2D images are presented along with the associated 1D central axis crossplane profile using the Monte Carlo method for example purposes. Note that the color ranges and EPID response scales are different between images.

### 3.1 Comparison of PSM

**FIGURE 2:**
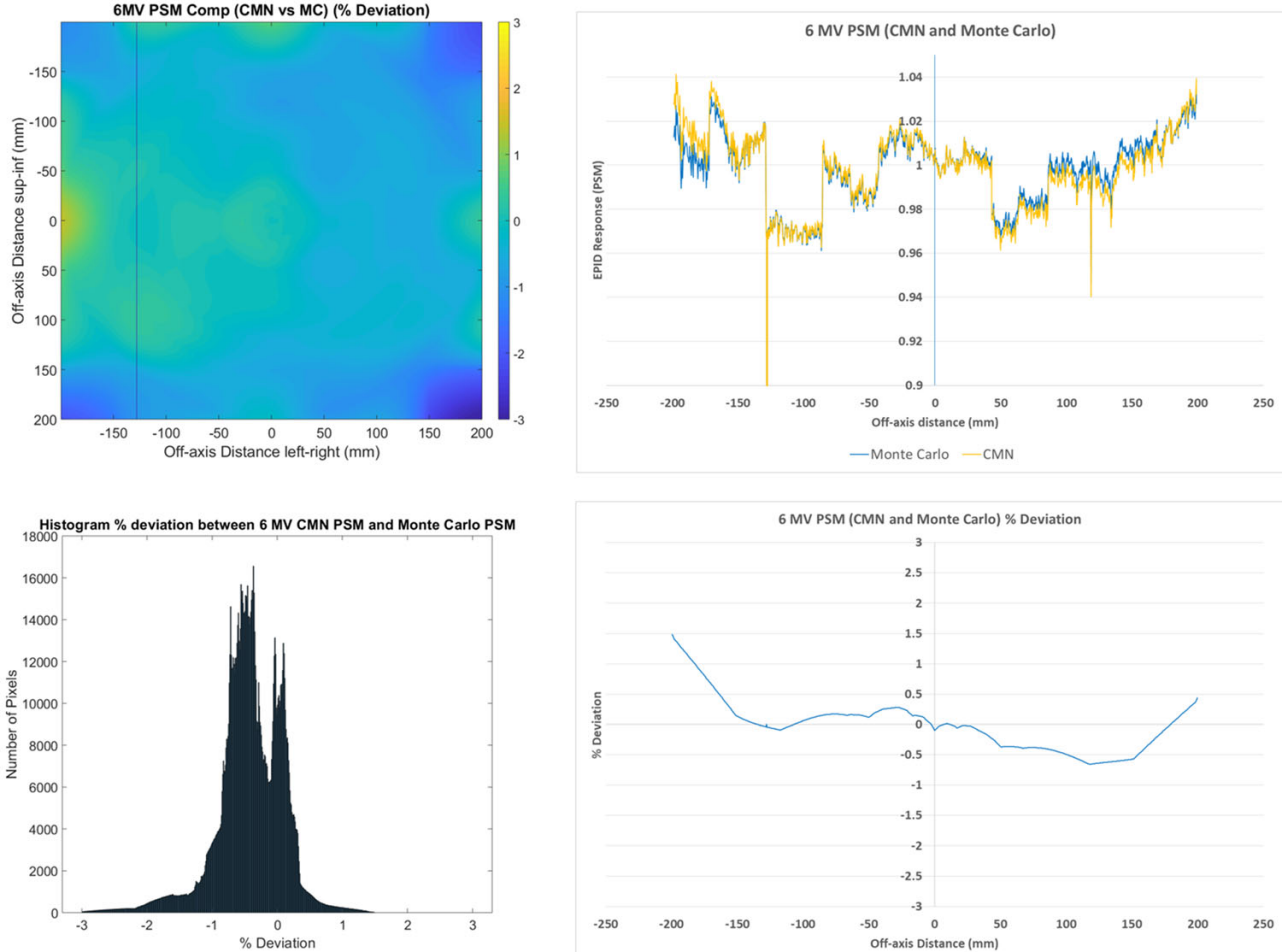
Comparisons of 6 MV Calvary Mater Newcastle (CMN) pixel‐sensitivity‐map (PSM) compared to Monte Carlo PSM presented as a 2D percentage deviation map (top left) and a corresponding percentage deviation histogram (*n* = 1190 × 1190 = 1 416 100) (bottom left) as well as 1D central axis crossplane profiles (top right) and percentage deviation along the crossplane profile (bottom right)

The CMN and Monte Carlo method results are compared in Figure 2. The PSMs from these methods agree to within ±1% for the majority of pixels. The mean agreement is ‐0.36%, indicating that the Monte Carlo PSM results in slightly higher pixel sensitivities. The median agreement is also ‐0.36% and the spread is 0.51% (1 SD). The percentage deviation map shows greater differences on one side of the image, which is likely due to the Monte Carlo method being idealized and not capturing the small asymmetry present in the beam and hence raw image, which in turn means that this asymmetry is erroneously being included in the PSM. It is also noteworthy that the column of dead pixels between ‐100 and ‐150 is correctly being included in the PSM rather than the Beam‐Response for both methods, however, the effect of the primary collimator, which technically should be included in the Beam‐Response is causing regions of low agreement in the corners of the image and hence may not be accounted well in the CMN method. The absence of high‐frequency noise in the 1D profile percentage deviation indicates that the Monte Carlo and CMN methods provide the same relative pixel sensitivity even if other features of the PSM may potentially be less well characterized. This is to be expected as the Beam‐Responses from both the CMN and Monte Carlo methods are inherently smooth so that the relative pixel sensitivities, which manifest as noise in the raw image, are wholly included in the PSM.

**FIGURE 3:**
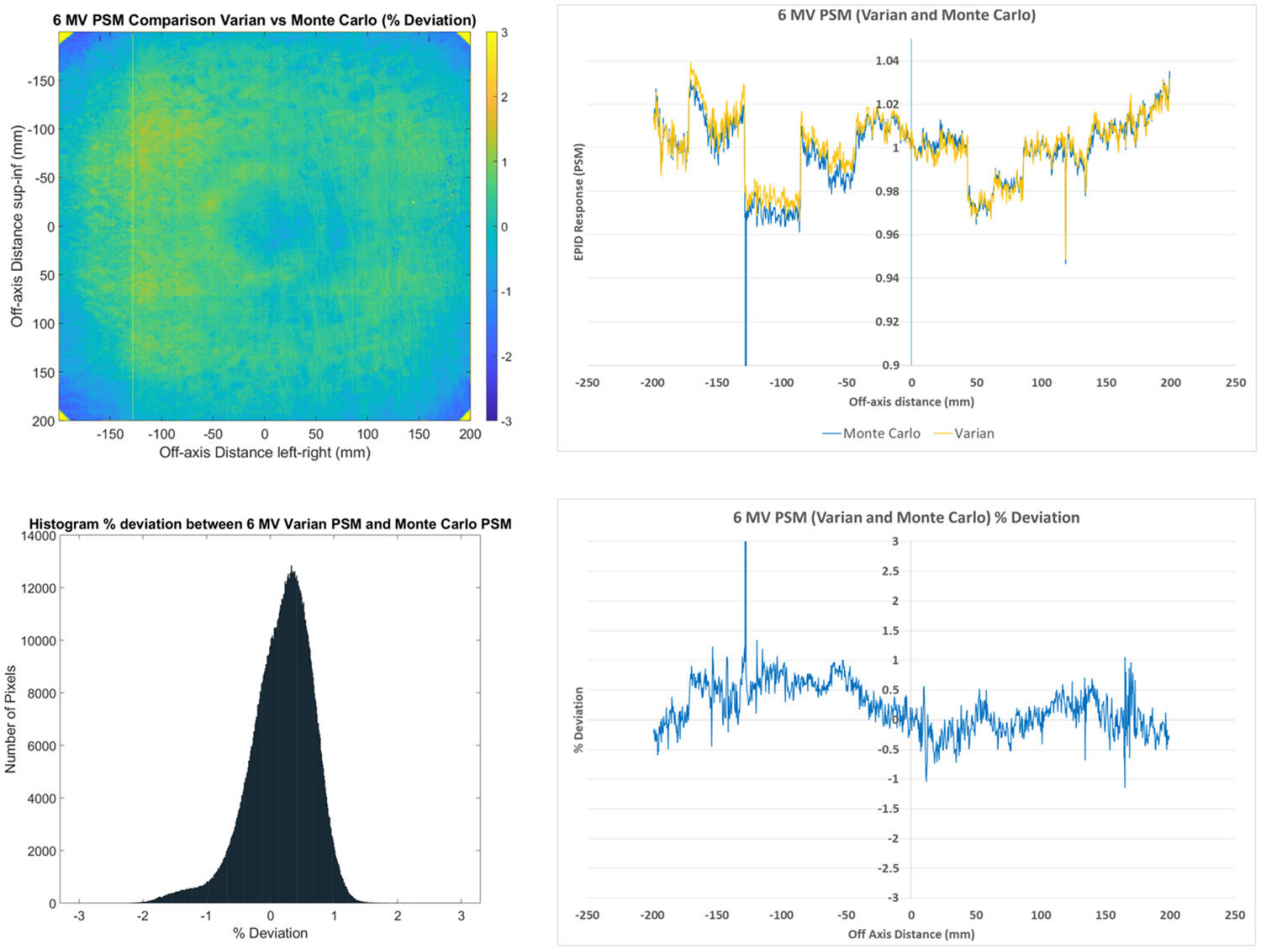
Comparisons of 6 MV Varian pixel‐sensitivity‐map (PSM) compared to Monte Carlo PSM presented as a 2D percentage deviation map (top left) and a corresponding percentage deviation histogram (*n* = 1190 × 1190 = 1 416 100) (bottom left) as well as 1D central axis crossplane profiles (top right) and percentage deviation along the crossplane profile (bottom right)

The Varian and Monte Carlo method results are compared in Figure 3. Agreement between the two methods is within ±1% for the majority of pixels. The mean agreement across the whole panel is 0.74% with a spread of 413% (1 SD). This mean value and wide spread are likely influenced by large magnitude differences at the extremities of the image where there are edge effects, in the corners where agreement is influenced by the presence of the primary collimator and by dead pixels, which in the Varian method appears to be included in the Beam‐Response rather than the PSM. By definition, dead pixels should be included in the PSM. In terms of general agreement between the results of the two methods the median agreement is likely a better metric than the mean. The median agreement is 0.24% and qualitatively the percentage deviation histogram is well centered about 0%, which is considered excellent.

The noisy percentage deviation 1D profile of Figure 3 indicates that the individual pixel sensitivities are not characterized correctly by the Varian method. This is expected to result in erroneous noise present in the Beam‐Response, which would likely require subsequent post‐processing to remove before the Beam‐Response could be analyzed for QA applications.

**FIGURE 4:**
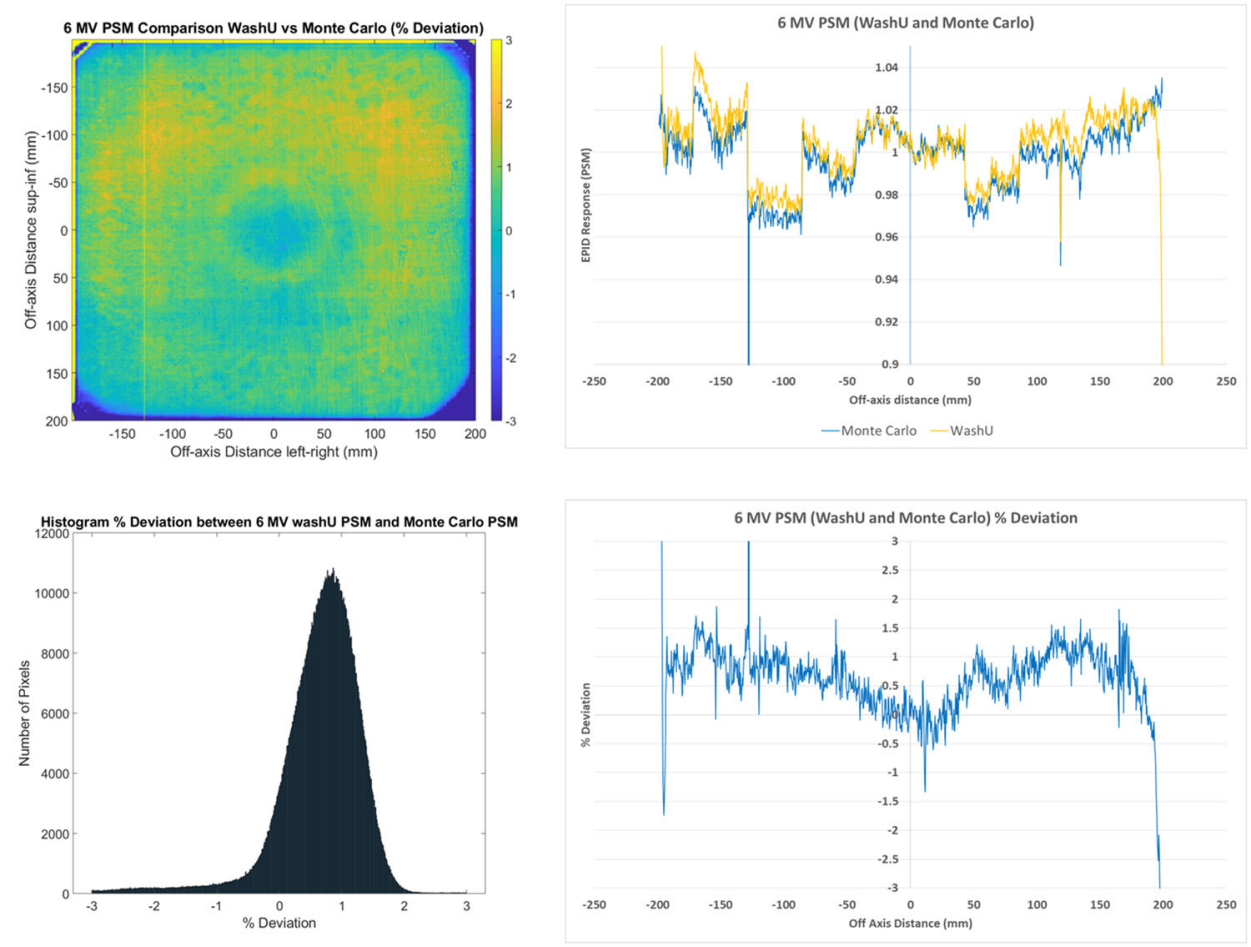
Comparisons of 6 MV WashU pixel‐sensitivity‐map (PSM) compared to Monte Carlo PSM presented as a 2D percentage deviation map (top left) and a corresponding percentage deviation histogram (*n* = 1190 × 1190 = 1 416 100) (bottom left) as well as 1D central axis crossplane profiles (top right) and percentage deviation along the crossplane profile (bottom right)

The WashU PSM and the Monte Carlo PSM results are compared in Figure 4, and are generally within 2%, with mean agreement at 1.1%. The regions beyond 2% agreement are at the edges and corners of the images, which are unlikely to be clinically significant. In these regions of the image the PSM cannot be derived accurately due to smaller field size (37 × 37 cm^2^) used in the delivery to avoid the irradiation of electronics. This results in the open fields not all overlapping in these regions with the penumbra of the beams providing a large uncertainty in the derivation of PSM. This, along with the inclusion of dead pixels in the Beam‐Response rather than the PSM like the Varian method, skews the mean result. The median agreement is measured to be 0.74%, which qualitatively appears to better represent the peak in the histogram. This 0.74% median agreement value and the apparent positive offset of the histogram indicate a general over estimation of pixel sensitivity of the WashU method compared to the Monte Carlo method. Since the PSM of all methods is normalized to the central 10 × 10 pixels during post‐processing then this should remove systematic variation. Therefore, the general overestimation of the WashU method compared to the Monte Carlo method must be a relative overestimation across the image. Similar to the Varian method the 1D percentage deviation profile appears noisy indicating inaccurate characterization of the pixel sensitivities which will cause the Beam‐Response to be noisy.

### 3.2 Comparison of Beam‐Response

**FIGURE 5:**
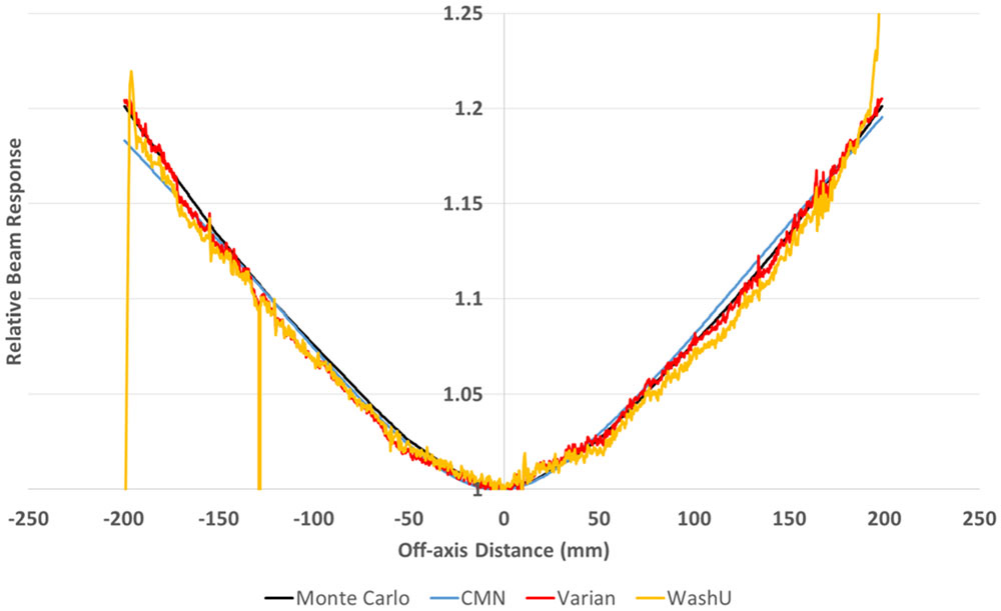
Crossplane central axis profile for the 6 MV Beam‐Responses measured with all four methods

Figure 5 shows the 1D central axis crossplane Beam‐Response profiles as determined by each method for the 6 MV beam. The general shape is qualitatively consistent between all methods, but the noisy Beam‐Response of the Varian and WashU methods are evident, as expected from the PSM results. There are inaccuracies evident toward the edges for the WashU method, as previously reported.^14^ The Monte Carlo method idealization of the Beam‐Response mentioned previously means that any drift in beam steering or non‐ideal beam energy is not represented and this is a potential source of discrepancy in Figure 5 between the Monte Carlo methods and the empirical methods.

### 3.3 PSM short‐term repeatability

**FIGURE 6:**
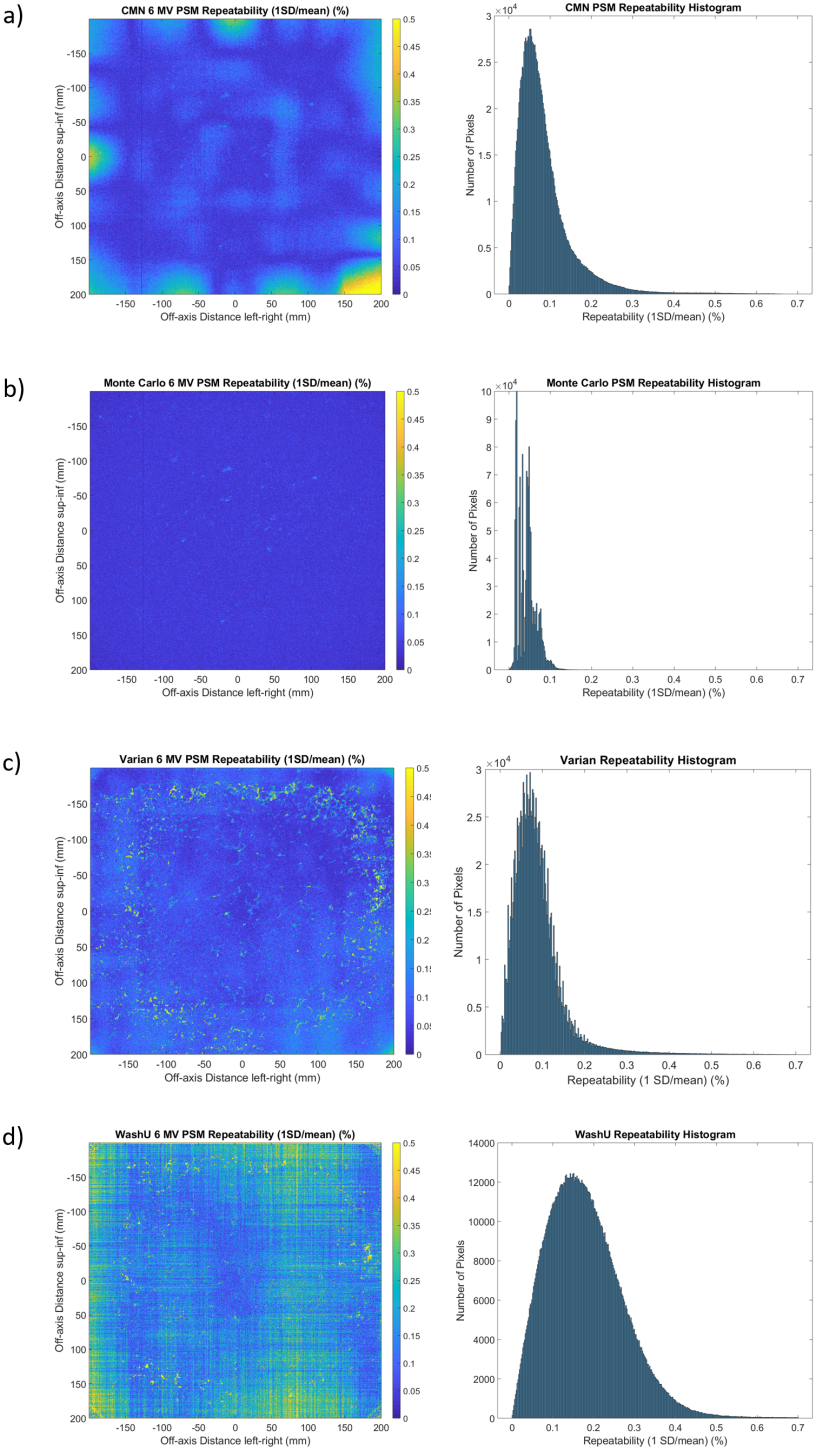
Pixel‐sensitivity‐map (PSM) repeatability for all methods (1 standard deviation [SD] %). (a) Calvary Mater Newcastle (CMN) method, (b) Monte Carlo method, (c) Varian method, and (d) WashU method. Two‐dimensional repeatability map (left) and corresponding repeatability histogram (*n* = 1190 × 1190 = 1 416 100) (right)

The results of Figure 6 show the short‐term repeatability (*n* = 3) for the PSM as determined by all methods. For the CMN method repeatability 95% of pixels are within 0.21%, with the median less than 0.1%. This is considered excellent repeatability. The CMN 2D repeatability map of Figure 6a (left) consistently shows repeatability within 0.5% and pixels outside this range are largely toward the edges and corners of the image, which are of least interest for QA applications. These areas of reduced repeatability could be due to issues in extrapolation used in the CMN method.

The Monte Carlo method PSM repeatability results, presented in Figure 6b, show excellent repeatability with 95% of pixels within 0.08%. This is the best repeatability of all methods tested. Because the Monte Carlo method determines the PSM by removing a consistent Monte Carlo calculated Beam‐Response from the raw image, the repeatability of the Monte Carlo method is really a measure of the repeatability of the raw image itself. Since the repeatability is better than any of the empirical methods then this would suggest that the empirical methods themselves are introducing additional variability. It is unclear whether the variability in the image itself is due to instability of the EPID panel or in the incident beam.

In the Monte Carlo method 2D repeatability map of Figure 6b (left) there are small areas with higher variability than their surroundings. This likely indicates pixel regions of relatively higher instability as such a phenomenon would unlikely be caused by instability in the beam, which would manifest more systematically. Such small areas are also visible in the CMN method repeatability results of Figure 6a and are also likely to be present in the other methods, but cannot be distinguished. This could potentially be an indicator of dying pixels.

The results of Figure 6c show the repeatability of the Varian measured PSM, with 95% of pixels within 0.19%. This is comparable to the CMN method. Unlike the CMN method the pixels of higher variability are not as co‐located, but spread more generally and individually, but with a bias toward the outer regions of the image.

The results of Figure 6d show the repeatability of the WashU method. This is inferior to the other methods, with 95% of pixels within 0.35%. This may be due to sensitivity of the method to small deviations in the EPID arm position. However, the vast majority of points show repeatability within 0.5%, which is still likely acceptable for QA applications.

### 3.4 Practicality of methods

The results of Table 1 show that the WashU method provides the quickest data acquisition and export times. The Varian method takes approximately twice as long, and the CMN method approximately 2.5 times as long. All methods require computational expertise and in this respect are considered similar.

**TABLE 1:** Time required to acquire and export data for each pixel‐sensitivity‐map (PSM) method (minute:second ± 1 SD)

Method	Time
WashU	7:9 ± 0:1
Varian	14:36 ± 0:5
CMN	17:48 ± 0:8

Abbreviations: CMN, Calvary Mater Newcastle; SD, standard deviation.

## 4 DISCUSSION

The definition of the PSM used in this study is that the PSM comprises the non‐uniformities introduced into the EPID image from the EPID panel itself, rather than from the beam. From this definition, it is clear that dead pixels should be included in the PSM rather than in the Beam‐Response. This is the case in the Monte Carlo and CMN methods, but not in the WashU and Varian methods. The appearance of the primary collimator in the corner of the image should be included in the Beam‐Response rather than the PSM. This is the case with the WashU and Varian methods, but not with the CMN and Monte Carlo methods, but could be rectified by changes to the fitting and extrapolation conditions in these regions.

Agreement with the Monte Carlo method is within 1% for the CMN and Varian methods and within 2% for the WashU method. However, while the Monte Carlo method has been used as reference in this study, it is not considered a gold standard and has inherent weaknesses. These include its idealization of the Beam‐Response in terms of beam symmetry and flatness, and the post‐processing and fitting applied to obtain a smooth Monte Carlo Beam‐Response from highly noisy calculations due to the small pixel size and difficulties in generating enough histories to reduce statistical noise in such small voxels. For a real clinical beam, as used in the empirical methods, it is likely that there are small, but clinically acceptable (<2%) asymmetries in the actual beam and potential slight energy differences from ideal that are not being modeled in the Monte Carlo Beam‐Response. When the Monte Carlo PSM is generated by removing the Monte Carlo Beam‐Response from a real open field raw image these small discrepancies in Beam‐Response from actual will translate into discrepancies introduced into the PSM.

Since the incident beam is considered to be smooth off‐axis then the Beam‐Response is also expected to be smooth. This is the case for the CMN and Monte Carlo methods, as expected, as both methods utilize smooth mathematical fitting functions to the Beam‐Response. However, both the WashU and Varian methods result in noisy Beam‐Responses, which suggests that some of the pixel sensitivity is not being fully characterized in the PSM and is hence being included in the Beam‐Response.

For the aS1200 EPID panel used in this study the inaccuracy of the WashU method at the extremities of the image is not likely to be significant as the extreme edges of the EPID panel are unlikely to be used in any QA application. However, if the methods were applied on an aS1000 EPID panel, which has a smaller imager size than the aS1200 then the effect may be significant in certain applications. The results of Figures 2 and 3 suggest that the CMN and Varian methods have superior performance to the WashU method toward the edge of the EPID panel.

The measured short‐term repeatability for all methods is demonstrated within 0.5%. The WashU method provided the worst repeatability. The results show that the incident beam and EPID response are highly repeatable and that all the PSM methods are repeatable to levels likely acceptable for QA applications. Considering that each of the empirical methods utilize EPID panel shifts then this suggests that the aS1200 EPID panel (at least the one used in this study) can be shifted both accurately and repeatably. The Monte Carlo repeatability measurements are free of method introduced variability. Considering that the Monte Carlo repeatability was highest of all methods then this suggests some added variability introduced by each of the empirical methods, but of magnitude that would likely be acceptable for QA applications.

The long‐term reproducibility of the PSM has not been assessed in this study, but has been addressed in other studies.^15^, ^29^ King et al.^29^ specifically examined the long‐term pixel stability of Varian aS500 EPID panels. They found that over a 3‐year period mean pixel variations were between 0.29% and 0.6%, and that more than 99% of all pixels showed variations less than 1%. In the study of Cai et al.,^15^ the WashU method was used to determine the PSM on the same linac 3 months apart. It was found that for the majority of pixels there was a less than 1% variation.

The time required to acquire and export data for each of the three empirical methods ranged from approximately 7 min for the WashU method up to 18 min for the CMN method. This can be considered an advantage for the WashU method, but the time required for any method would likely not be prohibitive in clinical practice.

All empirical methods require customized treatment plans or developer mode XML files to deliver, and custom software for analysis. In this respect of practicality all empirical methods are considered to be similar. The Monte Carlo method Beam‐Response need only be acquired once and then simply removed from an updated raw open field to obtain an updated PSM. However, due to the idealization for the Beam‐Response as discussed the generated PSM will only be accurate if the beam has been steered to optimal. Even then small discrepancies can be expected due to uncertainties in the beam steering process and user to user variations. The latter means that user variability is potentially added each time the PSM is updated, which is not present in the empirical methods. Also, since the Beam‐Response shape is energy dependent^14^ then for machines whose beam energy is acceptable, but not optimal a further error will be added into the PSM by using the Monte Carlo Beam‐Response. To explain this point further; according to Varian Installation Product Acceptance (IPA) procedures TrueBeam photon beams are considered energy matched if their percentage‐depth‐dose at 10 cm depth (PDD_10_) values agree with the nominal value to within ±0.5%.^30^ However, Yaddanapudi et al.^14^ found that for the 6 MV beam a change in PDD_10_ of 1% resulted in a 2.5% change in PSM‐corrected EPID flatness. This means that for two linacs at opposite extremes of acceptable beam energy (one at +0.5% PDD_10_ and one at ‐0.5% PDD_10_) then their EPID measured flatness will be 2.5% different to each other and approximately 1.25% different from nominal, and hence from the Monte Carlo Beam‐Response that has been calculated assuming ideal beam energy. This variation in Beam‐Response for a linac with acceptable, but not optimal beam energy will feed directly into the derived PSM and will be a source of error when the Monte Carlo method is used.

The aS1200 EPID is a 43 × 43 cm panel although only 40 × 40 cm is used for dosimetry mode and hence only 40 × 40 was investigated in this study. However, in the future there may be some application for using the full 43 × 43 cm panel in which case having the PSM for the whole panel would be required. All methods should theoretically be able to be modified for the full panel PSM. This is already available for the WashU and Varian methods, although the WashU method has inaccuracy at the EPID extremities as shown in this and previous studies.^14^

This study was performed on the aS1200 EPID with accompanying backscatter plate that successfully removes the backscatter contribution,^31^ and so EPID arm backscatter was not considered in this study. However, for the commonly used aS1000 EPID there is no backscatter shielding plate. For such EPIDs, the WashU method has been demonstrated to be accurate.^13^ While not proven, it is theorized that the Monte Carlo method would likely be best for properly characterizing the backscatter non‐uniformity in the PSM, followed by the WashU method. This is because the WashU method utilizes only small EPID panel shifts (4 mm shift) compared to the CMN and Varian methods, and hence backscatter is more consistent throughout the data collection process. However, due to the energy and field size dependence of the EPID arm backscatter the application of the PSM is non‐trivial and maybe better dealt with by separating the backscatter component from the PSM and dealing with the two separately.

## 5 CONCLUSIONS

Three empirical methods of PSM generation have been compared, primarily against the Monte Carlo method as reference, for a 6 MV beam, and their relative strengths and weaknesses identified and discussed. All methods are considered likely to be clinically acceptable with the choice of method most likely dependent on the needs of the specific application. The photon beam dependence of the PSM and of the four methods to measure it will be investigated in part 2 of this study.

## CONFLICTS OF INTEREST

Co‐authors Baozhou Sun, Bin Cai, and Matthew Schmidt report personal consulting fees and honoraria with Varian Medical Systems outside of the submitted work.

## AUTHOR CONTRIBUTIONS

Michael Barnes had the original idea for the study, performed data acquisition and analysis, and primarily wrote the manuscript. Baozhou Sun provided the WashU and Varian PSM methods and for these methods performed both post‐processing of data to generate PSM and wrote the relevant methods section of the manuscript. Baozhou also provided scientific input into the study and writing of the manuscript. Brad Oborn provided the Monte Carlo simulations, wrote the Monte Carlo‐related methods sections and provided scientific input into the study and writing of the manuscript. Bishnu Lamichhane performed the 2D curve fitting required for the CMN method and wrote the corresponding section in the methods as well as providing scientific input into the study and writing of the manuscript. Matthew Schmidt, Stuart Szwec, Bin Cai, Fred Menk, and Peter Greer provided scientific input into the study and writing of the manuscript.
